# Patient-Portal Compared with Supplemental In-Office Tablet Screening for Health-Related Social Needs in Primary Care

**DOI:** 10.1007/s11606-024-08929-x

**Published:** 2024-07-09

**Authors:** Kerry Meltzer, Max Yang, Alice Rossmann, Eliza W. Kinsey, Peter F. Cronholm, Anna U. Morgan

**Affiliations:** 1 National Clinician Scholars Program, Perelman School of Medicine, University of Pennsylvania, Philadelphia, PA USA; 2 Crescenz Veterans Affairs Medical Center, Philadelphia, PA USA; 3 Leonard Davis Institute of Health Economics, University of Pennsylvania, Philadelphia, PA USA; 4 College of Arts and Sciences, University of Pennsylvania, Philadelphia, PA USA; 5 Division of General Internal Medicine, Department of Medicine, Perelman School of Medicine, University of Pennsylvania, Philadelphia, PA USA; 6 Department of Family Medicine and Community Health, Perelman School of Medicine, University of Pennsylvania, Philadelphia, PA USA; 7 Center for Public Health, University of Pennsylvania, Philadelphia, PA USA

**Keywords:** health-related social needs, social determinants of health, patient-portal, health disparities

## Abstract

**Background:**

Screening for health-related social needs (HRSN) has become more widespread but the best method of delivering the screening tool is not yet known.

**Objective:**

Describe HRSN screening completion rate, specifically portal-based and in-person tablet-based screening.

**Design:**

Cross-sectional retrospective observational study.

**Participants:**

Adults age 18 or older who had a non-acute primary care visit at one of three internal medicine primary care clinics at a large, urban, academic medical center between July 2022 and July 2023.

**Main Measures:**

We identified the proportion of individuals who were screened using the HRSN questionnaire, whether screening was completed by patient-portal or tablet, as well as the degree of burden of HRSN. Using the electronic health record, we explored associations between sociodemographic characteristics and HRSN attributes.

**Key Results:**

Our study included 24,597 patients, of whom 37% completed the HRSN questionnaire. A smaller proportion of Black/African American patients and those with Medicaid insurance completed the questionnaire, yet they comprised a greater percentage of those who screened positive for unmet HRSN (*p* ≤ 0.001). Most patients completed the questionnaire by patient-portal (86.1%) compared with in-office tablets (14.0%). A larger proportion of those who completed screening by tablet screened positive for HRSN. Of all patients screened, 21.8% were positive for an unmet HRSN and 11.5% had more than one unmet HRSN.

**Conclusions:**

A majority of patients are not being screened for HRSN and results illustrate disparities when screening patients for HRSN through portal-based compared with supplemental in-office tablet-based screening. Prevalence of unmet HRSN varied by demographics such as race and insurance status.

**Supplementary Information:**

The online version contains supplementary material available at 10.1007/s11606-024-08929-x.

## INTRODUCTION

It is well known that socioeconomic conditions influence the health of individuals and communities.^1^ Adverse conditions including food insecurity, housing instability, transportation difficulties and unemployment—which together constitute health-related social needs (HRSN)—can contribute to negative health outcomes.^2,3^ In recent years, the Center for Medicare and Medicaid Services (CMS) has emphasized the importance of screening for HRSN, including adjusting payments for health systems according to social risk.^4,5^ Recognizing that unmet HRSN contribute to health disparities, The National Academies of Sciences, Engineering, and Medicine (NASEM) also recommends integration of social care into health care and increased research on the implementation of HRSN screening practices.

Prevalence of unmet HRSN across the United States has been consistently documented, and risk for screening positive in previous studies has been higher for Non-Hispanic Black patients and those using Medicaid.^6–9^ One study found that compared with private insurance, having Medicaid increased the odds of reporting a HRSN by two to eightfold, and Non-Hispanic Black individuals had a twofold higher odds of reporting a food or housing risk than White individuals.^9^ Fortunately, addressing HRSN in the medical context has been shown to improve patient-reported outcomes—including self-reported diet intake, resolution of unmet HRSN, overall well-being and patient satisfaction—along with improvements in population health—including in blood pressure, low-density lipoprotein cholesterol and medication adherence—and cost.^2,10^

While health systems have implemented patient-administered screening tools through electronic health record (EHR) portals, in-office through tablet or paper, or face-to-face with a medical assistant or provider, it is not yet known how successful these screenings tools have been in obtaining completed HRSN data.^11,12^ Although research has found that patients are generally supportive of social needs screening, several studies indicate that screening, especially for marginalized populations, may exacerbate stigma and discrimination.^12–14^ Electronic screening formats, as opposed to face-to-face screening, may allow patients to disclose more sensitive information, and previous studies have shown that individuals are open to completing questionnaires that are either portal or tablet-based.^13,15^

While portal-based screening tools increase efficiency of providers by reducing time spent on screening during office visits, research has shown that individuals who use patient portals are more likely to be White or Asian, younger, have private insurance, and have a higher annual income.^16,17^ Additionally, in a study evaluating completion of HRSN screening by patient-portal, patients with Medicaid insurance or who live in neighborhoods with a greater area deprivation index, a measure of neighborhood disadvantage,^18^ were less likely to complete the questionnaire.^9^ Thus, patient-portal screening may be missing some key populations who are more likely to screen positive for HRSN, prompting the need to evaluate the implementation of different screening methods. This study aims to fill this gap by examining the HRSN screening completion rate among patients screened via a portal-based tool prior to an in-office visit compared with patients who did not complete portal-based screening and instead were screened in-office at primary care internal medicine clinics at a large academic medical center.

## METHODS

We performed a cross-sectional retrospective study of adults age 18 and older who had an office-based non-acute primary care visit at one of three internal medicine primary care clinics at a large, urban, academic medical center from July 1, 2022 through July 1, 2023.

### HRSN Questionnaire

The HRSN questionnaire was developed using validated items^19^ to screen patients about exercise, stress, finances, education, food insecurity, transportation, alcohol, and housing instability (eAppendix) and was first implemented in the academic health system in July 2020. Given our health system’s focused efforts to address four HRSN in particular—financial insecurity, food insecurity, housing instability and transportation difficulties—based on selected CMS priorities^20^ and the health system’s social work response capacity, our analysis concentrated on the answers to these questions.^1^ Because only a portion of the questionnaire was used, the tool was not validated for the purposes of this study. Questions used in our analysis are italicized in eAppendix, with bolded answers indicating positive responses. For questions answered on a Likert scale, the HRSN was considered positive if the patient answered in the affirmative.

Patients were eligible for the standardized HRSN questionnaire if they had not completed it in the past 12-months. If patients had an active portal account, they received the survey through the patient-portal three days prior to a non-acute visit including annual visits or routine follow-ups. Participants were not offered incentives for completing the survey. In May 2022, the three clinics began using in-office tablets for patients to complete the survey if they had not done so via the portal prior to their office visit. If no tablet option was offered to a patient or was available—either because the tablet was not charged or malfunctioning—patients were not screened.

Upon arrival to their appointment, if a patient had not started the questionnaire, or had started but not completed the screening, front desk staff would be prompted to ask the patient to complete the questionnaire via tablet. The last question asked patients if they would like assistance with any HRSN that were identified in the questionnaire. If they responded “Yes,” an automatic referral was generated to social work if the patient screened positive for food insecurity. Questionnaire responses were available to the provider at the time of the office visit. Referrals to the clinic social worker for needs other than food insecurity were made at the discretion of the provider. The automatic referral for food insecurity only was a decision made by the care management infrastructure across more than 25 primary care practices based on social work capacity. After consulting with social workers at each clinic, primary care leadership determined that food insecurity was the most actionable of the needs identified and therefore was the first need to be included in the automated referral process.

### Data Collection and Analysis

Patient characteristics, including age, sex, race, ethnicity, primary insurance and address were identified using EHR data and measured in December 2023. Sex, race, and ethnicity were self-reported in the EHR. Given inconsistent collection of gender identity in the EHR, sex was analyzed rather than gender. Primary insurance was classified into the following categories: Private, Medicaid, Medicare, Self-Pay, or Tricare/VA. Data analysis was performed using Stata software version 17.^21^ We compared characteristics using chi-square tests for categorical variables and analysis of variation tests for continuous variables. A p-value of < 0.05 was used as a threshold for statistical significance. The study was deemed exempt by the University of Pennsylvania Institutional Review Board.

## RESULTS

From July 1, 2022 to July 1, 2023, 24,597 patients had at least one scheduled non-acute visit across the three internal medicine primary care offices (Table 1) and were therefore eligible for screening. The average age of patients was 53.7 years and patients were predominately female (61.3%). A majority of the patients identified as either Black/African American (53.4%) or White (34.1%) and 95% identified as Non-Hispanic. Most patients had private insurance (52.2%) as their primary payor while 18.1% had Medicaid and 27.8% had Medicare.

**Table 1:** Demographics of Patients Seen in Selected Internal Medicine Clinics from July 1 2022 – July 1 2023

Characteristics^*^	Total population *n* = 24,597	Screened *n* = 9,181	Not Screened *n* = 15,416	*p-*value
Age, mean (SD)	53.7 (17.5)	53.4 (17.1)	53.8 (17.7)	0.065
Sex				0.183
Female	15,069 (61.3)	5,680 (61.9)	9,389 (60.9)	
Male	9,525 (38.7)	3,499 (38.1)	6,026 (39.1)	
Race				≤ 0.001
Asian	1,371 (5.6)	623 (6.8)	748 (4.9)	
Black/African American	13,143 (53.4)	3,814 (41.5)	9,329 (60.5)	
White	8,379 (34.1)	4,171 (45.4)	4,208 (27.3)	
Other^†^ or Unknown	1,704 (6.9)	573 (6.2)	1,131 (7.3)	
Ethnicity				0.334
Hispanic or Latino	825 (3.4)	323 (3.5)	502 (3.3)	
Non-Hispanic or Latino	23,357 (95.0)	8,713 (94.9)	14,644 (95.0)	
Patient declined or Unknown	415 (1.7)	145 (1.6)	270 (1.8)	
Insurance				≤ 0.001
Private	12,830 (52.2)	5,341 (58.2)	7,489 (48.6)	
Medicaid	4,452 (18.1)	1,242 (13.5)	3,210 (20.8)	
Medicare	6,842 (27.8)	2,452 (26.7)	4,390 (28.5)	
Self-pay	428 (1.7)	124 (1.4)	304 (2.0)	
Tricare/VA	45 (0.2)	22 (0.2)	23 (0.2)	
Screening Type				
Portal		7,900 (86.1)		
Tablet		1,281 (14.0)		
Patients reporting HRSN^‡^		2,003 (21.8)		
Financial Insecurity		1,310 (14.3)		
Food Insecurity		1,118 (12.2)		
Housing		814 (8.9)		
Transportation		535 (5.8)		
No. of patients with HRSN				
1 HRSN		948 (10.3)		
2 HRSN		510 (5.6)		
3 HRSN		371 (4.0)		
4 HRSN		174 (1.9)		

^*^Characteristics were obtained through EHR-review and are displayed as *n* (%) unless otherwise specified

^†^Other includes American Indian or Alaskan Native, Native Hawaiian or Other Pacific Islander, or Some Other Race

^‡^*HRSN*,  Health-related social needs

### Comparing Patients Who Completed HRSN Questionnaire vs Those Who Did Not

Of these patients, 9,181 (37.0%) completed the HRSN questionnaire and 15,416 (63%) did not (Table 1). There was no significant difference between the age and sex of those who did and did not complete the questionnaire. There were significant differences in the proportions of racial groups and insurance categories. Compared to the total eligible population, Black/African American patients made up a smaller proportion of those who successfully completed HRSN screening (41.5% Black vs 45.4% White, *p* ≤ 0.001) and were overrepresented in the not screened population (60.5% Black vs 27.3% White, *p* ≤ 0.001). Similarly, while patients with private insurance and Medicaid represented 52.2% and 18.1% of the total eligible population respectively, 58.2% of patients who completed screening had private insurance and only 13.5% had Medicaid (*p* ≤ 0.001).

The majority of patients screened completed the questionnaire through the patient-portal (86.1%) while 14.0% completed the screening via tablets. Of the 1,281 patients who completed screening on the tablet, there were 788 (61.5%) who started the questionnaire at home and finished the screening in-office. Of the 15,416 patients who were never screened, 1,454 (9.4%) had started the questionnaire at home but never completed it in-office. Of all patients screened, 21.8% were positive for an unmet HRSN. The most common unmet HRSN cited was financial insecurity (14.3%) followed by food insecurity (12.2%), housing (8.9%), and transportation (5.8%). There were 1,055 patients (11.5%) who had more than one unmet HRSN.

### Comparing Patients Who Screened Positive vs Negative for HRSN

Table 2 presents the characteristics of patients who screened positive compared to those who screened negative for at least one of the four HRSN, which varied significantly by age, sex, race, insurance, and screening modality. Those who screened positive were younger on average (48.7 years vs 54.7 years for those who screened negative, *p* ≤ 0.001), and a greater proportion were female (69.3% among those who screened positive vs 59.8% among those who screened negative, *p* ≤ 0.001). A greater proportion of patients who screened positive were Black/African American (70.8%) than among those who screened negative (33.4%, *p* ≤ 0.001). The reverse was true for White patients where the proportion among those who screened positive was lower (18.7%) than among those who screened negative (52.9%, *p* ≤ 0.001). The proportion of those who received Medicaid was significantly higher among those who screened positive (33.6%) than among those who screened negative (7.9%, *p* ≤ 0.001). Although most patients completed screening through the portal, a greater proportion of those who screened negative for HRSN completed their screening through the patient-portal compared to those who screened positive (88.5% vs 77.2%, *p* ≤ 0.001). Instead, a greater proportion of patients identified with HSRN completed all or part of their screening using the in-office tablet compared to those who screened negative (22.8% vs 11.5%, *p* ≤ 0.001). For the distribution of HRSN, over half of patients who screened positive for HRSN reported financial insecurity (65.4%) or food insecurity (55.8%), while 40.6% reported difficulty paying for housing and 26.7% had transportation difficulties. More than half of patients who screened positive had more than one unmet HRSN and a greater proportion of these patients were Black/African American (75.6%) than among those who screened negative (33.4%, *p* ≤ 0.001).

**Table 2:** Patients Who Screened Positive and Negative for Unmet Health-Related Social Needs (HRSN)

Characteristics^*^	Screened Positive *n* = 2,003	Screened Negative *n* = 7,178	*p-*value
Age, mean (SD)	48.7 (15.8)	54.7 (17.3)	≤ 0.001
Sex			≤ 0.001
Female	1,387 (69.3)	4,293 (59.8)	
Male	615 (30.7)	2,884 (40.2)	
Race			≤ 0.001
Asian	69 (3.4)	554 (7.7)	
Black/African American	1,419 (70.8)	2,395 (33.4)	
White	375 (18.7)	3,796 (52.9)	
Other^†^ or Unknown	140 (7.0)	433 (6.0)	
Ethnicity			0.075
Hispanic or Latino	85 (4.2)	238 (3.3)	
Non-Hispanic or Latino	1,892 (94.5)	6,821 (95.0)	
Patient declined or Unknown	26 (1.3)	119 (1.7)	
Insurance			< 0.001
Private	834 (41.6)	4,507 (62.8)	
Medicaid	673 (33.6)	570 (7.9)	
Medicare	437 (21.8)	2,015 (28.1)	
Self-pay	55 (2.8)	69 (1.0)	
Tricare/VA	5 (0.3)	17 (0.2)	
Screening Type			≤ 0.001
Portal	1,547 (77.2)	6,353 (88.5)	
Tablet	456 (22.8)	825 (11.5)	
Patients reporting HRSN^‡^			
Financial Insecurity	1,310 (65.4)		
Food Insecurity	1,118 (55.8)		
Housing	814 (40.6)		
Transportation	535 (26.7)		
No. of patients with HRSN			
1 HRSN	948 (47.3)		
2 HRSN	510 (25.5)		
3 HRSN	371 (18.5)		
4 HRSN	174 (8.7)		

^*^Characteristics were obtained through EHR-review and are displayed as *n* (%) unless otherwise specified

^†^Other includes American Indian or Alaskan Native, Native Hawaiian or Other Pacific Islander, or Some Other Race

^‡^*HRSN*,  Health-related social needs

### Comparing Portal vs Tablet-Based Screening

Table 3 shows the characteristics of patients who completed screening via patient-portal compared with in-office tablets, which differed significantly by age, race, insurance, and unmet HRSN positivity. Those screened through the portal were younger, on average (50.2 vs 54.9 years, *p* = 0.001). A smaller proportion of patients who completed screening via the portal were Black/African American than among those who completed the screening via tablet (38.1% vs 62.8%, *p* ≤ 0.001). Conversely, the proportion of patients who completed screening via the portal who were White was higher than among those who completed the screening via tablet (48.3% vs 28.0%, *p* ≤ 0.001). The proportion of patients with private insurance was higher among those who screened negative for HSRN (59.7% vs 48.7%, *p* ≤ 0.001), while the inverse was true for patients with Medicaid (59.7% vs 48.7%, *p* ≤ 0.001). Although 19.6% of patients who completed portal-based screening were positive for HRSN, 35.6% of patients who completed tablet-based screening were positive (*p* ≤ 0.001). Prevalence of HSRN across all four categories was much higher among patients who completed the screening via tablet than among those who used the portal (*p* ≤ 0.001).

**Table 3:** Comparison of Patients Screened through Portal vs Supplemental Tablet

Characteristics^*^	Portal *n* = 7,900	Tablet *n* = 1,281	*p-*value
Age, mean (SD)	50.2 (16.9)	54.9 (16.8)	0.001
Sex			0.129
Female	4,856 (61.5)	824 (64.3)	
Male	3,042 (38.5)	457 (35.7)	
Race			≤ 0.001
Asian	570 (7.2)	53 (4.1)	
Black/African American	3,010 (38.1)	804 (62.8)	
White	3,813 (48.3)	358 (28.0)	
Other^†^ or Unknown	507 (6.4)	66 (5.1)	
Ethnicity			0.059
Hispanic or Latino	289 (3.7)	34 (2.7)	
Non-Hispanic or Latino	7,480 (94.7)	1,233 (96.3)	
Patient declined or Unknown	131 (1.7)	14 (1.1)	
Insurance			≤ 0.001
Private	4,717 (59.7)	624 (48.7)	
Medicaid	990 (12.5)	252 (19.7)	
Medicare	2,068 (26.2)	384 (30.0)	
Self-pay	104 (1.3)	20 (1.6)	
Tricare/VA	21 (0.3)	1 (0.1)	
Patients reporting HRSN^†^	1,547 (19.6)	456 (35.6)	≤ 0.001
Financial Insecurity	990 (12.5)	320 (25.0)	≤ 0.001
Food Insecurity	823 (10.4)	295 (23.0)	≤ 0.001
Housing	635 (8.0)	179 (14.0)	≤ 0.001
Transportation	417 (5.3)	118 (9.2)	≤ 0.001
No. of patients with HRSN			≤ 0.001
1 HRSN	765 (9.7)	183 (14.3)	
2 HRSN	376 (4.8)	134 (10.5)	
3 HRSN	276 (3.5)	95 (7.4)	
4 HRSN	130 (1.7)	44 (3.4)	

^*^Characteristics were obtained through EHR-review and are displayed as *n* (%) unless otherwise specified

^†^Other includes American Indian or Alaskan Native, Native Hawaiian or Other Pacific Islander, or Some Other Race

^‡^*HRSN*, Health-related social needs

## DISCUSSION

This study demonstrates gaps in care when integrating HRSN screening into the EHR workflow. Less than half of patients who were seen in one of three primary care clinics during the indexed time interval completed the questionnaire and a greater proportion of those who did were White and had private insurance. Also, while a large majority of patients who completed the questionnaire did so by patient-portal, a higher proportion of those who completed the questionnaire in person via tablet were Black/African American, had Medicaid insurance, and had more unmet HRSN. Thus, the study demonstrates the importance of the availability of multiple modalities for completing HRSN screening—including portal-based and in-office tablet-based screening—to reach the greatest number of patients with unmet HRSN and refer them to appropriate resources.

Our findings are similar to previous studies which have shown that Non-Hispanic Black patients and those with Medicaid have a higher degree of unmet HRSN and are also less likely to use the patient-portal.^6–9,16,17^ Furthermore, patients who have better health literacy and higher self-reported ability to use the internet are more likely to use online patient portals.^22,23^ Knowing that one in four Medicaid enrollees live in a home without internet or with limited computer access, this likely contributes to difficulty in accessing the questionnaire and the patient portal altogether.^24^ However, our overall response rate to HRSN screening is lower than what has been found in prior work, suggesting areas for significant improvement.^9,25^ Many patients in this study, despite not having completed the portal-based screening prior to their appointment, also did not complete in-office tablet-based screening at their visit. This study did not ask patients to identify why they did not complete the HRSN questionnaire either prior to or at their appointment, but there is likely a combination of factors that led to this. First, it may be that front desk staff did not prompt patients to complete the questionnaire, either because of failure of the prompting systems or because of time constraints where other issues or screenings took precedence. Second, clinics reported there were often technical problems with tablet-screening including tablets not being charged or malfunctioning, which meant tablets were often not available at time of patient check in. Third, patients may have declined the offer of tablet-based screening, which we did not have a means of capturing but which we are planning on exploring with front desk staff who distribute tablets. Future studies can help to expand our understanding of these key problems. Regardless, it is necessary that each clinic has a consistent workflow in place and has adequate infrastructure to ensure tablets are available and functioning for patient use. It’s also important that the survey is formatted in a way that is accessible to patients (e.g., easy to navigate with large readable font and appropriate contrast).^26^ For patients who did complete the HRSN screening, whether by portal or tablet, our study showed high rates of unmet HRSN which may be reflective of the population of patients seen in our practices.^9^

This study reinforces the need for improved HRSN screening, especially given the high proportion of patients with unmet HRSN particularly among Black/African American patients, as well those with Medicaid. Our findings may also influence how HRSN screenings are implemented across the health system, and encourage other health systems to incorporate a multi-modal portal plus tablet-based approach. A more comprehensive understanding of HRSN burden among patients may incentivize increased investment in social workers and partnerships with community-based organizations to connect patients with specific resources. Some best practices identified to increase screening and referral rates for HRSN have included utilization of standardized questionnaires, community health workers to connect patients to social services, and toolkits for clinician education.^27^

This study has several limitations. First, because the two screening tools were not randomized, as patients were only prompted to use the tablet if they had not completed the HRSN survey prior to their appointment, we cannot definitively attribute lower screening rates to either approach. Furthermore, because there is a differential distribution of patient characteristics between those screened using either tool and those not screened, this suggests there are potential unobserved confounders or barriers beyond screening through patient-portal which needs to be addressed. To better understand the success rates of each modality, a future study could randomize patients to receive the screening via portal or tablet. Second, the questionnaire was only administered in English. Given that previous studies have found that Non-English speaking patients are less likely to complete HRSN screening, and that Spanish-speaking patients have higher rates of unmet HRSNs, we may be missing key populations with high HRSN burden.^7,8^ Third, it is unknown how patients’ HRSN relate to their burden of comorbidities. Given that previous research has shown that screening rates are higher for patients who have a higher comorbid disease burden, this may be important to evaluate in future studies.^28^ Fourth, patient demographics were collected from the system’s EHR in December 2023, so demographics such as age, primary insurance, and address may have changed from when HRSN screening occurred. Fifth, we were unable to determine whether referrals sent to office social workers were completed and thus how patients’ unmet HRSN were addressed. This would be an important assessment to prioritize in future studies. Sixth, the study did not assess other screening approaches (e.g., paper forms or oral survey) which could potentially improve data capture among more vulnerable populations. Finally, because patients were not screened in-office by methods other than by tablet, it is unknown how many patients did not complete the questionnaire because of tablet unavailability, likely contributing to lower screening rates. The difficulty in establishing a consistent workflow for tablet-use underscores the importance of having staff trained in navigating workflow issues and that each clinic is equipped with adequate tablets for patient-use. Future studies could also consider staff-administered screening, which was not offered due to limited staffing capacity.

## CONCLUSION

We found that the HRSN questionnaire did not reach most patients seen at one of three primary care internal medicine clinics in our health system. A lower proportion of patients who are known to be at higher risk for poor health outcomes completed the questionnaire, which may be reflective of disparities found when screening is presented over patient-portal. This study supports a multi-modal approach to HRSN screening, including more robust use of in-office tablets, to ensure that patients who may have a higher degree of unmet HRSN are not missed.

## Supplementary Information

Below is the link to the electronic supplementary material.

Supplementary file1 (DOCX 33 KB)

## Data Availability

The data are not publicly available due to their containing information that could compromise the privacy of research participants.
