# Supplementary material for: Patient-Portal Compared with Supplemental In-Office Tablet Screening for Health-Related Social Needs in Primary Care
Source: J Gen Intern Med. 2024 Jul 9;39(13):2425–31. doi: 10.1007/s11606-024-08929-x (PMC11436592; doi:10.1007/s11606-024-08929-x)
Supplement: Supplementary file 1 — Supplementary file1 (DOCX 33 KB) [file 11606_2024_8929_MOESM1_ESM.docx]

**eAppendix. Penn Medicine’s standardized social needs screening survey**

We want to give you the best services and care possible to meet all of your needs. Your choice to answer or to not to answer any questions will NOT impact your ability to get care at Penn Medicine. In many cases, your answers will help us find out if there are any programs or supports that can give you added benefits and care. We cannot promise help in all areas, but will do our best to find ways we can help based on your priorities. Please let us know if you have any questions, concerns, or thoughts.

1. On average, how many days per week do you engage in moderate to strenuous exercise (like a brisk walk)? [0 days, 1 day, 2 days, 3 days, 4 days, 5 days, 6 days, 7 days, decline]
2. On average, how many minutes do you engage in exercise at this level? [0 minutes - .... - 150 or more minutes, decline]
3. Do you feel stress - tense, restless, nervous, anxious, or unable to sleep at night because your mind is troubled all the time - these days? [Not at all, Only a little, To some extent, Rather much, Very much, Decline]
4. *How hard is it for you to pay for the very basics like food, housing, medical care, and heating? [Not hard at all, Not Very Hard,* ***Somewhat hard, Hard, Very hard****, Decline]*
5. What is the highest level of school you have completed or the highest degree you have received? [1st grade, 2nd grade, 3rd grade, 4th grade, 5th grade, 6th grade, 7th grade, 8th grade, 9th grade, 10th grade, 11th grade, 12th grade, GED or equivalent, Associate degree: occupational, technical, or vocational, Associate degree: academic program, Bachelor’s degree (e.g., BA, AB, BS), Master’s degree (e.g., MA, MS, MEng, MEd, MSW, MBA), Professional school degree (e.g., MD, DDS, DVM, JD), Doctorate, Some college, no degree, Never attended school, Decline]
6. *Within the past 12 months, you worried that your food would run out before you got the money to buy more [Never true,* ***Sometimes true, Often True****, Decline]*
7. *Within the past 12 months, the food you bought just didn’t last and you didn’t have money to get more [Never true,* ***Sometimes true, Often True****, Decline]*
8. *In the past 12 months, has lack of transportation kept you from medical appointments or from getting medications? [****Yes****, No, Decline]*
9. *In the past 12 months, has lack of transportation kept you from meetings, work, or getting things needed for daily living? [****Yes****, No, Decline]*
10. How often do you have a drink containing alcohol? [Never, Monthly or less, 2-4 times a month, 2-3 times a week, 4 or more times a week, Decline]
11. How many drinks containing alcohol do you have on a typical day when you are drinking? [1 or 2, 3 or 4, 5 or 6, 7 to 9, 10 or more, Decline]
12. How often do you have six or more drinks on one occasion? [Never, Less than monthly, Monthly, Weekly, Daily or almost daily, Decline]
13. In the last 12 months, was there a time when you were not able to pay the mortgage or rent on time? [Yes, No, Decline]
14. In the last 12 months, how many places have you lived? [Free text entry]
15. *In the last 12 months, was there a time when you did not have a steady place to sleep or slept in a shelter (including now)? [****Yes****, No, Decline]*

If you identified a need with any of the areas included in this questionnaire, would you like assistance? [Yes, No, Decline]
